# Thermoregulatory Responses to Graded Exercise Differ among Sasang Types

**DOI:** 10.1155/2015/879272

**Published:** 2015-06-02

**Authors:** Duong Duc Pham, Jeong Hoon Lee, Eun Seok Park, Hyun Sung Baek, Ga Yul Kim, Young Boum Lee, BonCho Ku, Jong Yeol Kim, Chae Hun Leem

**Affiliations:** ^1^Department of Physiology, Ulsan College of Medicine, 388-1 Poongnap-dong, Songpa-gu, Seoul 138-736, Republic of Korea; ^2^Korea Institute of Oriental Medicine, 461-24 Jeonmin-dong, Yuseong-gu, Daejeon 305-811, Republic of Korea

## Abstract

We compared sweat rate and variables such as workload (*W*_*e*_), metabolic heat production (*H*_prod_), and temperature increment load (*T*_inc_) across Sasang types. 304 apparently healthy participants aged 20–49 years with their Sasang type determined were enrolled. Local sweat rates on the chest (LSR_chest_) and back (LSR_back_) were measured using a perspiration meter during a maximum treadmill exercise test. Oxygen uptake was measured continuously using a breath-by-breath mode indirect calorimeter. The TaeEum (TE) type had a larger body size, a higher percent body fat, and a lower body area surface area (BSA) to body mass compared with the other Sasang types, particularly the SoEum (SE) type. The TE type tended to have a shorter exercise time to exhaustion and lower maximal oxygen uptake (mL·kg^−1^·min^−1^) than the other types. LSR_chest_ in TE types was greater than that of the SE and SoYang (SY) types in men, whereas LSR_back_ was higher in the TE type than that of the other types in women. After normalizing LSR for *W*_*e*_, *H*_prod_, *T*_inc_, and BSA, this tendency still remained. Our findings suggest that the thermoregulatory response to graded exercise may differ across Sasang types such that the TE type was the most susceptible to heat stress.

## 1. Introduction

During exercise, 75% of metabolic energy is released as heat, causing body core temperature to rise. Thermoregulatory mechanisms maintain body temperature primarily through convection to the surrounding air and evaporation of sweat [1, 2]. Anthropometric characteristics such as body size, body surface area- (BSA-) to-mass ratio (BSA/M), percent body fat, and subcutaneous adipose tissue affect thermoregulation during exercise [3–5].

Sasang constitutional medicine (SCM) is a form of traditional Korean medicine that focuses on the relationship between phenotypes and clinical features. According to this theory, humans can be classified into four constitutional types, TaeYang (TY), SoYang (SY), TaeEum (TE), and SoEum (SE), which differ in their physiological and psychological features, vulnerability to particular patterns of illnesses, and responses to external conditions including the thermoregulatory response [6, 7]. According to SCM theory, sweat is an important sign of health states and determinant of human constitution. For instance, the TE type tends to sweat readily, and profuse sweating indicates a healthy state, whereas profuse sweating in the SE type, which is less prone to sweating, is the sign of an unhealthy state [6]. Several studies have shown that Sasang types have constitution-specific physical, psychological, and genetic characteristics [8–11]. Several self-report studies of sweating have found that the TE and SY types tend to sweat more than the SE type under the same environmental conditions [12, 13]. However, no studies have used objective and reliable methods to examine systematically thermoregulation and sweating capacity across Sasang types. We used an indirect calorimeter and hygrometer to objectively examine constitution-specific sweat responses during graded exercise to investigate the effect of anthropometric features on thermoregulation among Sasang types.

## 2. Methods

### 2.1. Participants

A total of 550 apparently healthy volunteers (297 males and 253 females) between the ages of 20 and 49 years were recruited through advertisements at the Asan Medical Center in Seoul City, the capital of Republic of Korea, between 2009 and 2012. The study was approved by the Institutional Review Board of the Asan Medical Center and informed consent was obtained from all participants.

### 2.2. Classification of Sasang Type

We used the Sasang Constitution Analysis Tool (SCAT) developed by the Korea Institute of Oriental Medicine (KIOM) to determine Sasang type. The SCAT has been described in detail previously [14]. Briefly, the SCAT identifies Sasang type using a multinomial logistic regression analysis based on the integrated data of facial features (two-dimensional images), body shape (width and circumference), voice features, and questionnaire responses (personality traits and physiological symptoms). The accuracy of the SCAT has been reported to be higher than that of the Questionnaire for Sasang Constitution Classification II, the most commonly used diagnostic tool for Sasang typing [14, 15]. Because the TY type is rare among Koreans, this version of the SCAT diagnosed only the TE, SE, and SY types.

### 2.3. Exclusion Criteria

Using the integrated data, the SCAT assessed the probability of each Sasang type for each participant (e.g., TE = 0.53, SE = 0.40, and SY = 0.13). We excluded individuals whose highest Sasang score was <0.40 and those in whom the difference between the highest and middle scores was <0.05 because their Sasang type was ambiguous (*n* = 237). The strict exclusion criteria were used to increase the reproducibility of the SCAT. The remaining 313 participants were classified according to their highest Sasang score, and their chest and back sweat rate were measured simultaneously during exercise. Further nine participants were excluded because they had unusual patterns of sweating (the increase in sweating was not steady and showed an abrupt increase and decrease during the early phase of exercise) or their oxygen consumption was not measured. Thus, the data of 304 participants (178 males and 126 females) were included in the analysis.

### 2.4. Resting and Exercise Oxygen Consumption

Participants were instructed to refrain from stimulants (smoking, alcohol, and coffee), heavy exercise, and eating a meal for 12 h prior to the experiment. Oxygen consumption (VO_2_) 1 min before (VO_2pre_) and during exercise (VO_2ex_) was measured using a breath-by-breath mode indirect calorimeter (Vmax ENCORE 29c—SensorMedics, VIASYS HealthCare, Yorba Linda, CA, USA). The VO_2_ measurement protocol has been described elsewhere [16]. Briefly, steady state VO_2pre_ was measured while participants were seated on a chair, and VO_2ex_ was measured while participants ran on a treadmill following a standard Bruce protocol and were verbally encouraged to continue until exhaustion. VO_2pre_ and VO_2ex_ were recorded at 20-s intervals and the average-per-minute measurements were used for further analyses. Furthermore, maximum oxygen consumption (VO_2max_, mL·kg^−1^·min^−1^), a measure of cardiorespiratory fitness, was recorded.

### 2.5. Workload, Heat Production, and Estimated Temperature Increment

Vertical displacement (kg·m·min^−1^) was calculated by multiplying the percentage grade (%) by speed (m·min^−1^) and body weight (kg). External workload (*W*_*e*_, Watt·min) was calculated as vertical displacement divided by 6.12 [17]. Total *W*_*e*_ was the sum of *W*_*e*_ per minute during exercise.

Metabolic energy expenditure (*M*_ee_, Watt·min) for each minute before and during exercise was calculated from the respiratory quotient (RQ) and VO_2_ using a modified equation described previously [18, 19], in which

(1) MeeWatt·min=VO2Litter×RQ−0.70.3×21.13+1−RQ0.3×19.62×16.67.

Metabolic heat production (*H*_prod_, Watt·min) for each minute of exercise was calculated by subtracting *W*_*e*_ from *M*_ee_. The total *H*_prod_ was the sum of *H*_prod_ per minute during exercise. Metabolic efficiency (%) was calculated as the percentage of *W*_*e*_ to *M*_ee_.

Because 1 kilocalorie (69.78 Watt·min) of energy expenditure is required to raise 1 kilogram of body water by 1°C [17] and *M*_ee_ before exercise refers to the resting *M*_ee_ required to maintain normal body temperature, the temperature increment load (*T*_inc_, °C) for each is

(2) Tincin degree Celsius=HprodWatt·min−Mee  befor exerciseWatt·min69.78×Body water kg.

### 2.6. Local Sweat Rate Measurement

Local sweat rate (LSR) at the chest (LSR_chest_) and back (LSR_back_) was measured using a perspiration meter (SKN 2000, Nishizawa Electric Meters Manufacturing Co., Ltd, Nagano, Japan) that used a ventilated capsule to estimate LSR based on (i) the difference in vapor content between effluent and influent air passing through a given skin area and (ii) the air flow rate adjusted to the levels of sweating and varied in a range between 300 and 600 mL·min^−1^ [20]. Two capsules were attached to the skin of the chest and back on the median line at the nipple level and using an adhesive band during the treadmill test. The treadmill exercise and sweat rate measurements were conducted at an ambient temperature of 23°C ± 1°C and 60% humidity. Because the capsule covered a 1-cm^2^ area of the skin, LSR was reported as mg per min per centimeter squared (mg·cm^−2^·min^−1^) at 20-s intervals. LSR_chest_ and LSR_back_ were measured continuously during exercise, and the average-per-minute values were used in the analysis. Whole-body sweat rate (WSR) at the chest (WSR_chest_) and back (WSR_back_) was calculated as LSR_chest_ and LSR_back_ multiplied by BSA (cm^2^), respectively. In a further analysis, sweat-induced factors were normalized by dividing LSR and WSR by *W*_*e*_, *H*_prod_, and *T*_inc_. The increment in sweat rate (SR) per each minute of exercise was calculated by subtracting the SR measured at the previous minute. The analysis was performed for LSR and WSR.

### 2.7. Body Composition Measurement

Body fat mass (BFM), fat-free mass (FFM), and body water were measured by bioimpedance analysis using the InBody 720 (Biospace, Seoul, Korea). The device uses a segmental multifrequency impedance technique, and its body composition measurements are comparable to those obtained using dual-energy X-ray absorptiometry (DXA), the standard body composition analyzer [21]. BSA was calculated using the formula of D. Du Bois and E. F. Du Bois [22]. Body weight and height were measured using a digital scale.

### 2.8. Data Analysis

All statistical analyses were conducted according to sex using R 3.1.2 for Windows. One-way analysis of variance (ANOVA) was used to evaluate differences in the demographic characteristics, body composition, cardiorespiratory fitness, exercise time, total *W*_*e*_, total *M*_ee_, and metabolic efficiency across Sasang types. Tukey's HSD test was used for post hoc comparisons.

The time-dependent analysis could not be performed using a repeated measures test because the exercise times differed among participants; thus, the Kruskal-Wallis rank sum test was used to assess differences in the mean rank of *W*_*e*_, *M*_ee_, *T*_inc_, and SR values for each minute of exercise. Significant differences were followed up using the Nemenyi test for post hoc pairwise multiple comparisons of ranked data using package PMCMR [23]. *P* values < 0.05 were deemed to indicate statistical significance.

## 3. Results

### 3.1. Demographic Characteristics, Anthropometric Indices, and VO_2max_

There was no age difference among Sasang types in men, whereas the TE women were older than SY women (*P* < 0.05). Conversely, height did not differ among Sasang types in females, whereas TE type males were taller than those in the SE group. The body size of the TE type males was larger than that of the other types and, as a consequence, body weight, BMI, BFM, FFM, BSA, and body water content were higher. The body size-linked values of the SE group were lower than those of the TE and SY types. Similarly, TE type females had a larger body size and higher body weight, BMI, BFM, FFM, BSA, and body water content than those in the SY and SE groups, although the body size-linked values did not differ between the SE and SY types.

VO_2max_ was highest among SY type males and lowest among those in the TE group, whereas VO_2max_ was comparable between females in the SY and SE types and lowest in the TE group (Table 1).

### 3.2. Exercise Time, *W*_*e*_, *M*_ee_, and *T*_inc_

**Figure 1:**
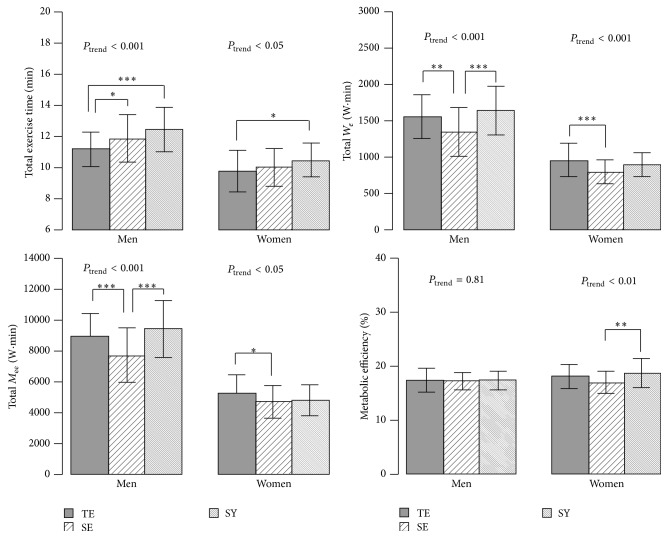
Total exercise time, external workload (*W*_*e*_), metabolic energy expenditure (*M*_ee_), and metabolic efficiency (*W*_*e*_ divided by *M*_ee_%) across Sasang types by gender. Data are presented as mean (SD). Sasang types include TaeEum type, SoEum type, and SoYang type. *P*_trend_ calculated by one-way ANOVA test. Tukey's HSD post hoc test: ^*∗*^ *P* < 0.05, ^*∗∗*^ *P* < 0.01, and ^*∗∗∗*^ *P* < 0.001.

Despite a similar total *W*_*e*_ (post hoc test, *P* = 0.34 in males and *P* = 0.21 in females) and an equal total *M*_ee_ in males (post hoc test *P* = 0.81), the exercise times of TE type males and females were shorter than those of the SY type males (11.1 ± 1.1 versus 12.4 ± 1.4 min, resp.; *P* < 0.001) and females (9.8 ± 1.3 versus 10.4 ± 1.1 min, resp.; *P* < 0.05). In contrast, the comparison between TE and SE type males revealed that SE males had a lower total *W*_*e*_ (post hoc test, *P* < 0.01) and lower total *M*_ee_ (post hoc test, *P* < 0.05) over a longer exercise period (post hoc test *P* < 0.05) than did TE males. Exercise time did not differ between the TE and SE type females, although the TE type females had a higher *W*_*e*_ than did SE females. The only difference in metabolic efficiency across Sasang types was that observed between SE and SY type females (Figure 1).

**Figure 2:**
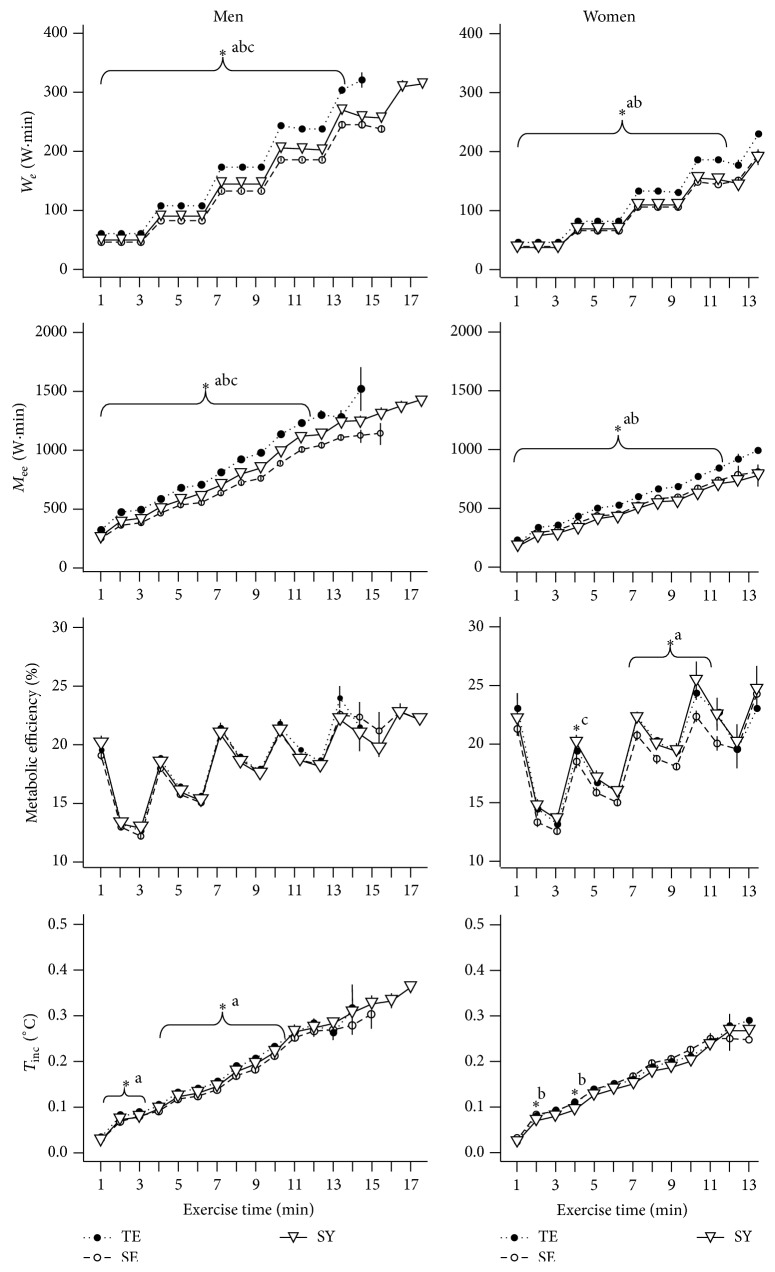
External workload (*W*_*e*_), metabolic energy expenditure (*M*_ee_), metabolic efficiency (*W*_*e*_ to *M*_ee_%), and temperature increment load (*T*_inc_) across Sasang types by gender. Data are presented as mean (SEM). TE, TaeEum type; SE, SoEum type; SY, SoYang type. ^*∗*^Significant difference between groups at each time point (*P* < 0.05) by Kruskal-Wallis rank sum test. Nemenyi's post hoc test: ^a^TE differs from SE; ^b^TE differs from SY; ^c^SE differs from SY.

Figure 2 shows that *W*_*e*_, *M*_ee_, metabolic efficiency, and *T*_inc_ increased during exercise. In males, the *W*_*e*_ and *M*_ee_ were significantly different among Sasang types at each exercise minute such that the values were highest in the TE and lowest in the SE type individuals. No differences in metabolic efficiency were found among Sasang types. TE type males had a higher *T*_inc_ between 2 and 10 min of exercise than did those in the SE group. In females, *W*_*e*_ and *M*_ee_ were higher in the TE type than in the SY and SE type individuals; however, these values did not differ between the SY and SE types. TE type females tended to have higher metabolic efficiency than those in the SE group; however, the difference was statistically significant at only a few exercise time points. With the exception of the early stage of exercise, the *T*_inc_ did not tend to differ among Sasang types in females.

### 3.3. Local and Whole-Body Sweat Rate during Exercise

**Figure 3:**
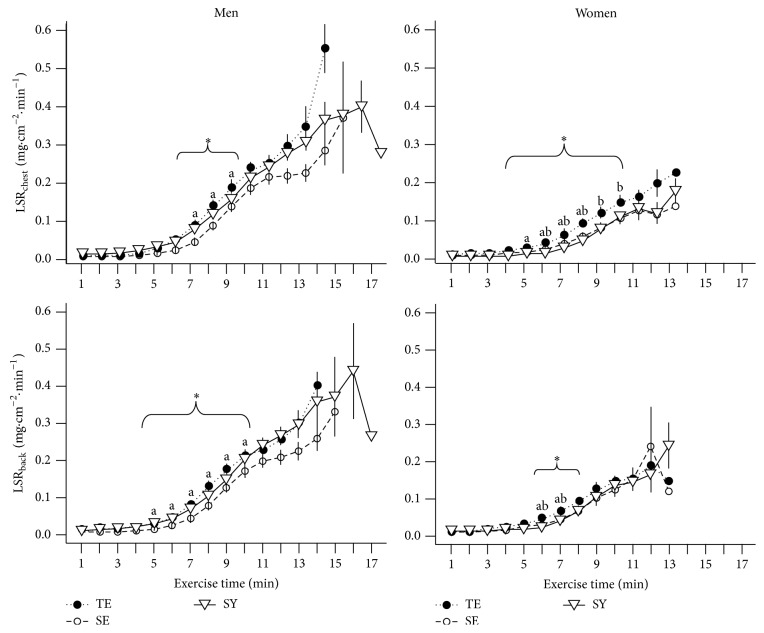
Local sweat rate measured on the chest (LSR_chest_) and on the back (LSR_back_) across Sasang types by gender. Data are presented as mean (SEM). TE, TaeEum type; SE, SoEum type; SY, SoYang type. ^*∗*^Significant difference between groups at each time point (*P* < 0.05) by Kruskal-Wallis rank sum test. Nemenyi's post hoc test: ^a^TE differs from SE; ^b^TE differs from SY; ^c^SE differs from SY.

**Figure 4:**
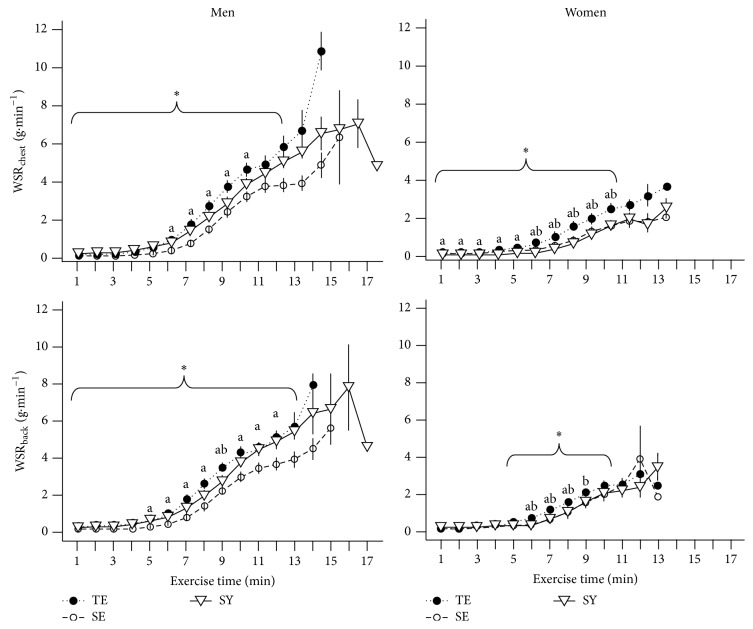
Whole-body sweat rate estimated by multiplying body surface are with LSR_chest_ (WSR_chest_) and with LSR_back_ (WSR_back_) across Sasang types by gender. Data are presented as mean (SEM). TE, TaeEum type; SE, SoEum type; SY, SoYang type. ^*∗*^Significant difference between groups at each time point (*P* < 0.05) by Kruskal-Wallis rank sum test. Nemenyi's post hoc test: ^a^TE differs from SE; ^b^TE differs from SY; ^c^SE differs from SY.

Figure 3 shows LSR_chest_ and LSR_back_ for each minute during the treadmill exercise across Sasang types according to sex. We found no difference in LSR values during the early stage of exercise; however, LSR_chest_ and LSR_back_ were higher in the TE than in the SY and SE types after 7 and 5 min, respectively, in males and after 5 and 6 min, respectively, in females. The LSR differences between Sasang types were ambiguous during the late stage of exercise because few TE and SE individuals were able to continue exercise for 17 min. We found a similar pattern in WSR: there were no differences in WSR_chest_ and WSR_back_ among Sasang types during the early stage of exercise, with the exception that TE females had a higher WSR_chest_ than did SE females from 1 min, followed by a significant increase in WSR_chest_ and WSR_back_ in TE type individuals compared with those in the SY and the SE group in particular, during the middle stage (Figure 4).

### 3.4. Sweat Rate Normalized to Sweat-Induced Factors during Exercise

**Figure 5:**
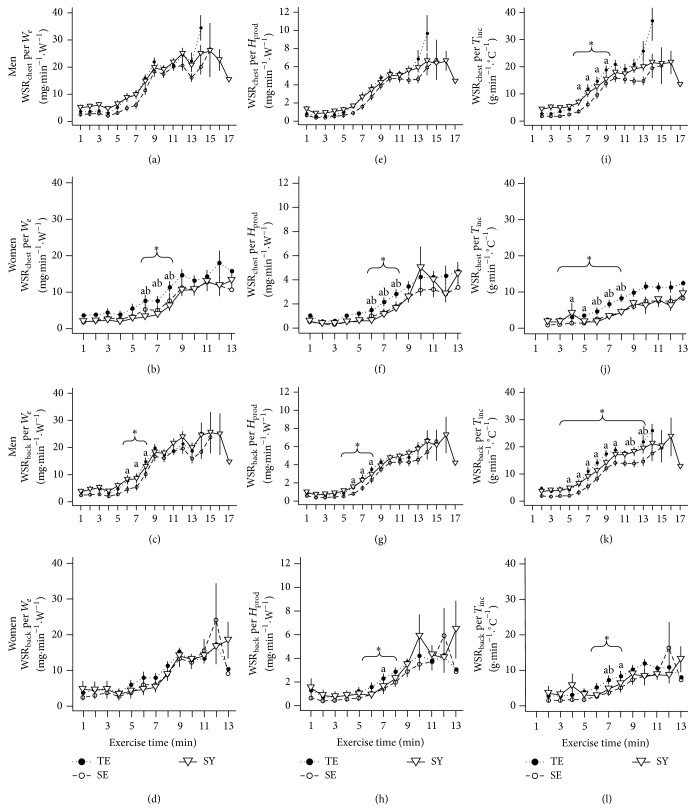
Whole-body sweat rate estimated from LSR_chest_ and LSR_back_ divided by external workload (WSR_chest_ per *W*_*e*_ and WSR_back_ per *W*_*e*_), by metabolic heat production (WSR_chest_ per *H*_prod_ and WSR_back_ per *H*_prod_), and by temperature increment (WSR_chest_ per *T*_inc_ and WSR_back_ per *T*_inc_) across Sasang types by gender. Data are presented as mean (SEM). TE, TaeEum type; SE, SoEum type; SY, SoYang type. ^*∗*^Significant difference between groups at each time point (*P* < 0.05) by Kruskal-Wallis rank sum test. Nemenyi's post hoc test: ^a^TE differs from SE; ^b^TE differs from SY; ^c^SE differs from SY.

We found no differences among Sasang types in WSR_chest_ normalized to *W*_*e*_ and *H*_prod_ in males (Figures 5(a) and 5(e)) and in WSR_back_ normalized to *W*_*e*_ and *H*_prod_ in females (Figures 5(d) and 5(h)). However, the TE type tended to show a greater increase in WSR_back_ normalized to *W*_*e*_ and *H*_prod_ in males and WSR_chest_ normalized to *W*_*e*_ and *H*_prod_ in females (Figures 5(c), 5(g), 5(b), and 5(f), resp.). WSR_chest_ and WSR_back_ normalized to *T*_inc_ were higher in TE type than in the SY, and the SE individuals in particular, during the middle stage of exercise (Figures 5(i), 5(j), 5(k), and 5(l)).

Analysis of the per-min increment in WSR normalized to *W*_*e*_, *H*_prod_, and *T*_inc_ revealed that, compared with the other types, TE males showed a greater increase in WSR_back_ normalized to *W*_*e*_ and *H*_prod_ at 6 and 7 min of exercise, whereas in females, WSR_chest_ and WSR_back_ normalized to *W*_*e*_ and *H*_prod_ were higher in the TE than other Sasang types. Normalization of WSR to *T*_inc_ revealed a consistent elevation of SR in the TE type individuals at the 6 and 7 min of exercise (see Figure S1 available online at http://dx.doi.org/10.1155/2015/879272). Furthermore, this pattern was observed in the LSR_chest_ and LSR_back_ data (Figures S2 and S3).

## 4. Discussion

Our study is the first to investigate the SR produced by graded exercise in Sasang typology. Our primary finding was that the SR was highest among TE and lowest among SE participants during the middle stage of graded exercise, and this difference persisted when SR was normalized to sweat-induced factors such as *W*_*e*_, *H*_prod_, and *T*_inc_. We also found that the *T*_inc_ was higher in the TE type during exercise, particularly in men, and that the TE type had a shorter total exercise time to exhaustion than other Sasang types.

Individual variations in anthropometric characteristics, such as BSA, BSA/M, adipose tissue thickness, and intensity of the exercise, have been shown to affect the thermoregulatory response. Evidence indicates that smaller body size, regardless of training status, can endure a greater maximal physical workload [24, 25]. Yokota et al. [26, 27] found that core temperature during physically related heat stress was likely to be higher in males and females with high percent body fat than in lean individuals. This may be because smaller individuals have a high BSA/M, which is a distinct thermal advantage for heat loss, and the homeostatic imbalance caused by heat retention during exercise is controlled more efficiently. The significant amount of heat generated by exercise may be a crucial factor in the reluctance of heavier individuals to engage in prolonged physical activities [28, 29]. The anthropometric characteristics of the Sasang types in our study (i.e., larger body size, higher percent of fat mass, and a lower BSA/M in the TE compared with the other types and small body size, low fat mass percentage, and high BSA/M in the SE individuals) were comparable with those reported previously [16, 30]. Although we did not measure the change in body temperature during exercise, the estimated *T*_inc_ based on oxygen consumption showed the greatest increase in the TE type participants. Increase in *T*_inc_ during graded exercise may induce a higher SR, a lower VO_2max_, a proxy for physical fitness, and a shorter time to exhaustion in the TE type.

Because the TE type participants had a higher BMI and body fat percentage than did the SY and SE types, the subcutaneous adipose tissue, a layer of impeding thermal convection, may have been thicker in the TE type individuals. Moreover, the relative surface for heat convection, the BSA/M, was smaller in TE type participants; thus, heat loss was conducted primarily through evaporation by sweating rather than convection via the skin-environment thermal gradient. Recently, Kim et al. [31] reported that the TE type subjects had higher viscoelasticity and lower elasticity hysteresis at the forearm than did the SE type individuals. Further studies are needed to investigate the constitution-specific differences in convection and radiation activity and skin blood flow during exercise.

Several studies have shown that sweating sensitivity in individuals native to tropical regions, who have smaller, thinner bodies than those native to temperate climate zones, was relatively lower than that of temporal region natives [32, 33]. This physiological trait may be a crucial factor in the protection of tropical natives from heat stress, particularly during prolonged physical activity. Interestingly, the response to heat stress in short-term heat adapted individuals is related to an increase in the amount of sweat produced, whereas long-term heat acclimation was associated with the economical use of body fluid [30]. Thus, it is reasonable to suggest that the potential differences in thermoregulatory responses among Sasang typologies may be due to inherited long-term heat acclimation in which sweating sensitivity may play an important role. We did not examine sweat gland function in the present study and further investigation is warranted.

Different body sizes among the Sasang types were associated with differences in *W*_*e*_ and *H*_prod_ as skin surface areas differed. In theory, participants who performed at the same work intensity produced the same amount of heat and released the same total amount of sweat, whilst those with a higher BSA have a lower LSR despite producing the same total amount of sweat. Thus, Cramer et al. [18] proposed that LSR be compared under the same work intensity/heat production per square of BSA. Our analyses of the absolute LSR, LSR normalized to sweat-induce factors (*W*_*e*_, *H*_prod_, and *T*_inc_), and WSR all showed that the graded exercise-induced increase in SR was greater in TE type participants than in those of the other Sasang types.

Our findings should be interpreted in the context of several limitations. We assessed the LSR, which does not reflect whole-body evaporation. Furthermore, we consider this to be a pilot study because we examined only the macrophenomenon related to the sweating response and did not investigate the pattern of relevant variables such as body core and skin temperature and the distribution and activation of sweating glands. Thus, we were unable to provide a mechanistic explanation for the distinct sweating response patterns we observed among the constitutions.

In conclusion, our findings indicate that the thermoregulatory response to graded exercise may differ across Sasang types such that the TE type was the most susceptible to heat stress induced by prolonged exercise. Several TE-specific traits including high body fat mass, low BSA-to-mass ratio, thicker subcutaneous adipose tissue, and lower aerobic fitness may cause earlier exhaustion, earlier sweating onset, and a greater increase in sweat production during the treadmill exercise in TE type individuals compared with the other Sasang types.

## Supplementary Material

Supplementary material includes (1) Figure S1: Increment per minute of whole sweat rate (WSR) per external work load (W_e_) , WSR per metabolic heat production (H_prod_) , and WSR per temperature increment load (T_inc_) across Sasang types by gender, (2). Figure S2: Local sweat rate (LSR) measured on the chest and on the back divided by W_e_, by H_prod_, and by T_inc_ across Sasang types by gender, and (3) Figure S3: Increment per minute of LSR per W_e_ , LSR per H_prod_ , and LSR per T_inc_ across Sasang types by gender. We found a consistent tendency that the TaeEum type had a higher elevation of sweat rate in comparison with that in other Sasang types at 6 and 7 min of exercise in all figures from S1 to S3.

## Figures and Tables

**Table 1:** Demographic characteristics, anthropometric indices, and maximal oxygen consumption according to Sasang type.

	TE	SE	SY	*P*
Males				
*n*	62	59	57	
Age (yrs)	29.8 (6.1)	30.1 (6.3)	29.8 (6.2)	0.95
Weight (kg)	82.6 (9.4)^a, b^	62.8 (6.4)^c^	70.8 (6.4)	<0.001
Height (cm)	174.9 (5.5)^a^	172.3 (5.3)	173.5 (5.5)	0.02
BMI (kg·m^−2^)	27.0 (2.6)^a, b^	21.2 (1.8)^c^	23.5 (1.6)	<0.001
BSA (m^2^)	1.98 (0.13)^a, b^	1.74 (0.10)^c^	1.85 (0.09)	<0.001
BSA/M (cm^2^·kg^−1^)	241.1 (13.0)^a, b^	278.9 (14.9)^c^	261.1 (9.9)	<0.001
Fat-free mass (kg)	61.6 (5.9)^a, b^	52.2 (4.7)^c^	57.5 (4.5)	<0.001
Body fat (kg)	21.1 (6.5)^a, b^	10.6 (3.4)^c^	13.3 (4.0)	<0.001
Body water (kg)	45.1 (4.3)^a, b^	38.4 (3.5)^c^	42.2 (3.3)	<0.001
VO_2max_ (mL·kg^−1^·min^−1^)	532.7 (97.1)^a, b^	739.2 (121.0)^c^	682.8 (121.3)	<0.001
Females				
*n*	46	40	40	
Age (yrs)	31.9 (6.7)	30.6 (7.1)	28.1 (5.2)	0.02
Weight (kg)	64.0 (7.2)^a, b^	50.9 (4.3)	52.5 (4.0)	<0.001
Height (cm)	159.9 (5.1)	161.3 (5.2)	159.0 (5.7)	0.18
BMI (kg·m^−2^)	25.1 (2.7)^a, b^	19.6 (1.5)^c^	20.8 (1.6)	<0.001
BSA (m^2^)	1.66 (0.10)^a, b^	1.52 (0.08)	1.53 (0.08)	<0.001
BSA/M (cm^2^·kg^−1^)	261.6 (15.5)^a, b^	299.3 (12.1)^c^	291.1 (11.0)	
Fat-free mass (kg)	42.5 (4.0)^a, b^	37.9 (3.6)	38.3 (3.7)	<0.001
Body fat (kg)	21.5 (5.2)^a, b^	13.1 (3.1)	14.2 (2.8)	<0.001
Body water (kg)	31.1 (3.0)^a, b^	27.8 (2.7)	28.1 (2.7)	<0.001
VO_2max_ (mL·kg^−1^·min^−1^)	546.1 (114.5)^a, b^	758.2 (136.8)	716.5 (129.2)	<0.001

Data are expressed as means (SD). BMI, body mass index; BSA, body surface area; BSA/M, body surface area per body weight; VO_2max_, maximal oxygen consumption during the treadmill exercise. Sasang typology: TE, Taeum type; SE, Soeum type; SY, Soyang type. *P* values were calculated using one-way analysis of variance. Tukey's HSD post hoc test: ^a^TE differed from SE; ^b^TE differed from SY; ^c^SE differed from SY.
