# Supplementary figures and images for: Thermoregulatory Responses to Graded Exercise Differ among Sasang Types
Source: Evid Based Complement Alternat Med. 2015 Jun 2;2015:879272. doi: 10.1155/2015/879272 (PMC4468316; doi:10.1155/2015/879272)

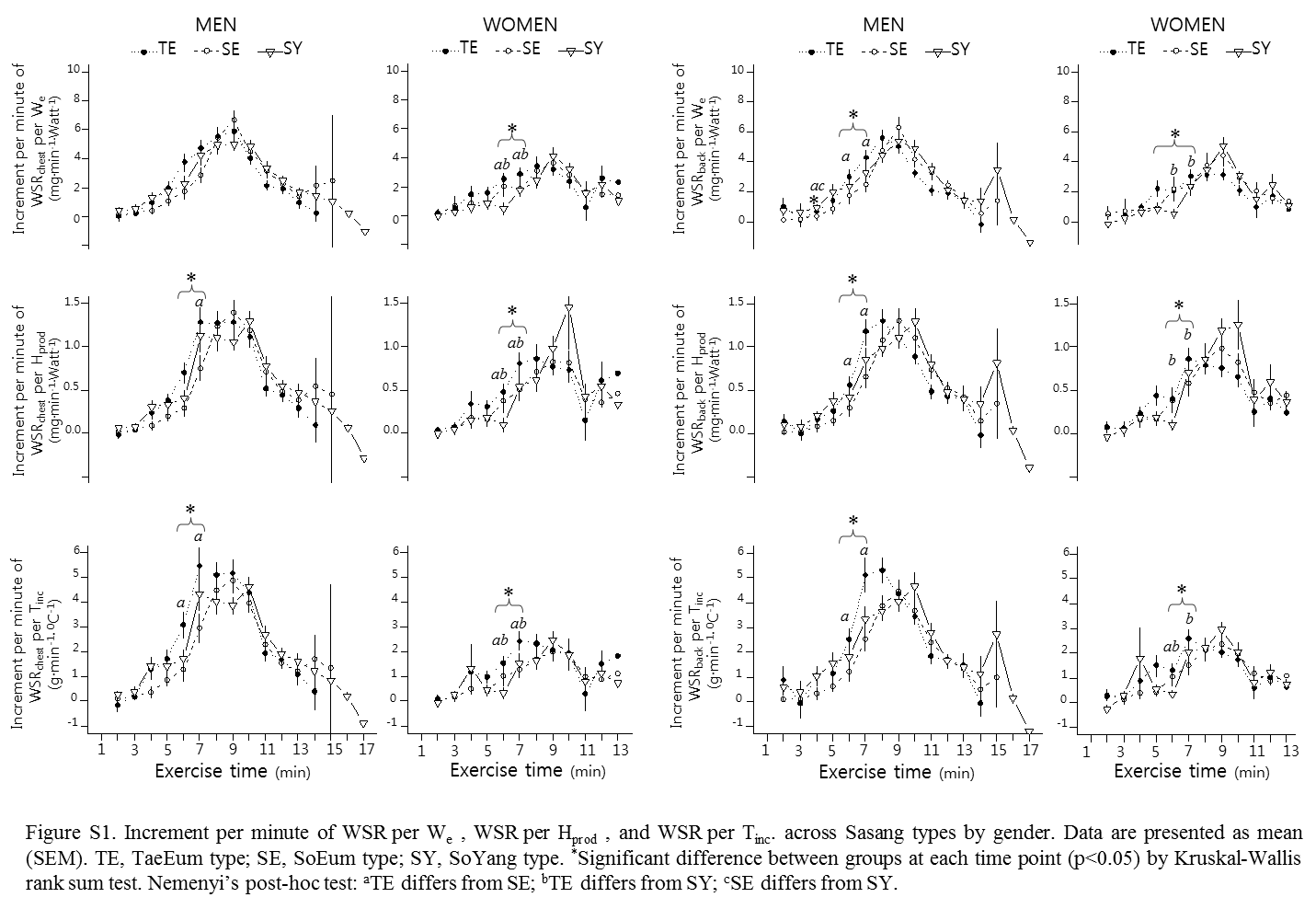


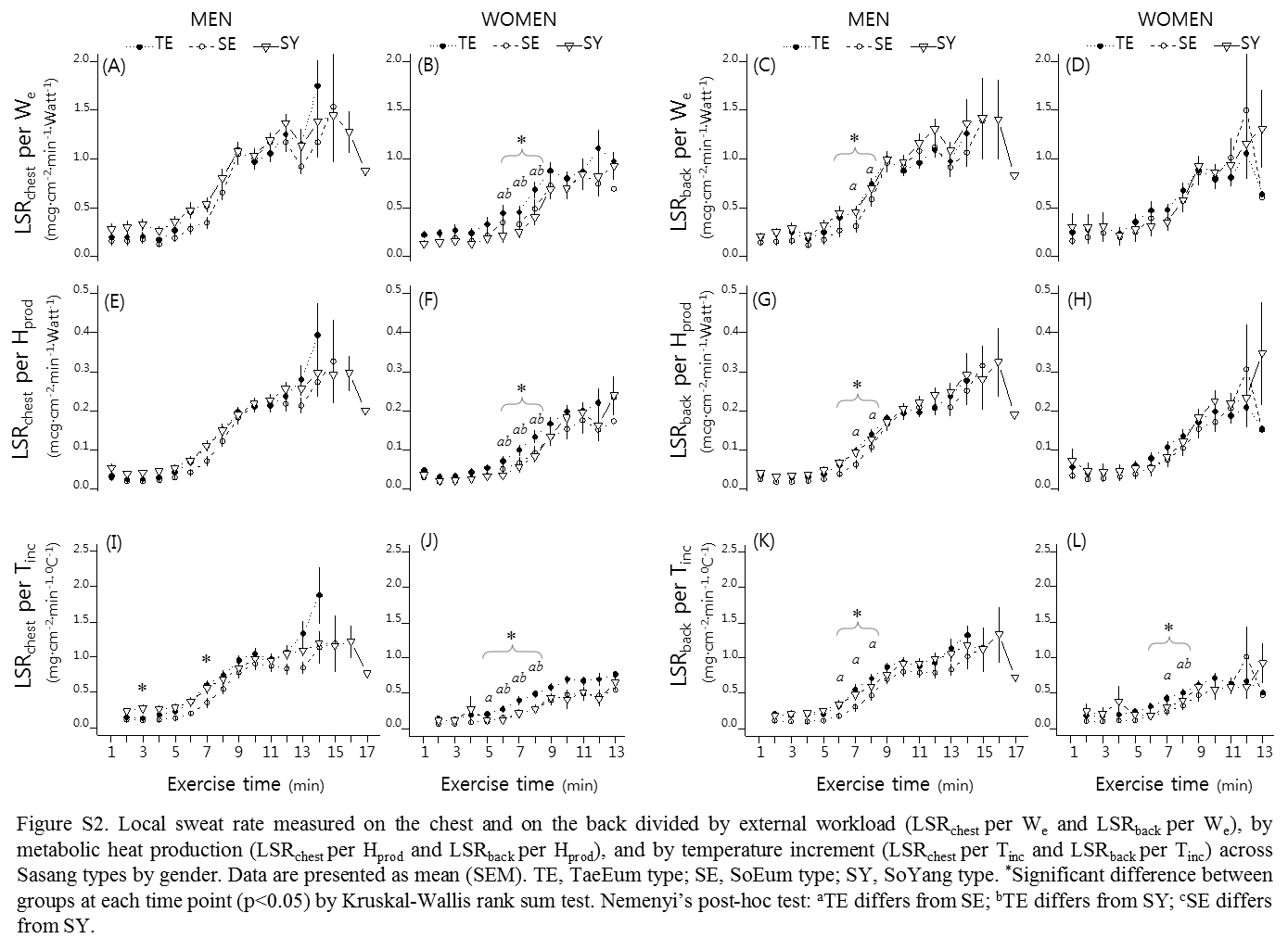


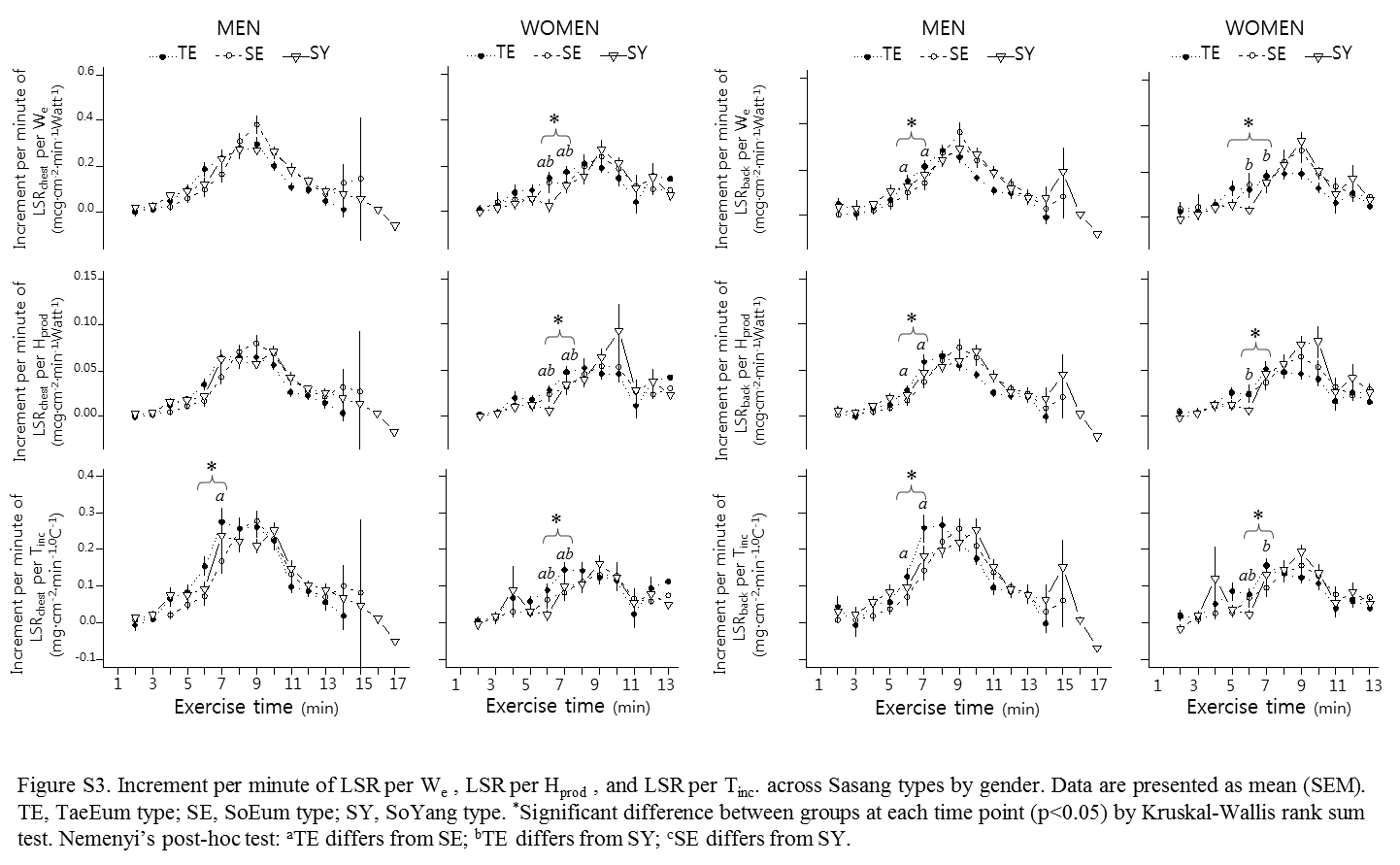

Supplement: Supplementary file 1 — Supplementary material includes (1) Figure S1: Increment per minute of whole sweat rate (WSR) per external work load (We) , WSR per metabolic heat production (Hprod) , and WSR per temperature increment load (Tinc) across Sasang types by gender, (2). Figure S2: Local sweat rate (LSR) measured on the chest and on the back divided by We, by Hprod, and by Tinc across Sasang types by gender, and (3) Figure S3: Increment per minute of LSR per We , LSR per Hprod , and LSR per Tinc across Sasang types by gender. We found a consistent tendency that the TaeEum type had a higher elevation of sweat rate in comparison with that in other Sasang types at 6 and 7 min of exercise in all figures from S1 to S3. [file 879272.f1.doc]
